# Reinforcement of Bonding Strength and Water Resistance of Soybean Meal-Based Adhesive via Construction of an Interactive Network from Biomass Residues

**DOI:** 10.3390/polym11060967

**Published:** 2019-06-03

**Authors:** Zhiwei Chang, Huiwen Pang, Anmin Huang, Jianzhang Li, Shifeng Zhang

**Affiliations:** 1 MOE Key Laboratory of Wooden Material Science and Application, Beijing Forestry University, Beijing 100083, China; changzhiwei222@163.com (Z.C.); panghuiwen1010@bjfu.edu.cn (H.P.); lijzh@bjfu.edu.cn (J.L.); 2 Beijing Key Laboratory of Wood Science and Engineering, Beijing Forestry University, Beijing 100083, China; 3 Research Institute of Wood Industry, Chinese Academy of Forestry, Beijing 100091, China; hbham2000@sina.com

**Keywords:** soybean meal-based adhesive, natural fiber-reinforced, filler, hydrogen bonding, crosslinked structure, bonding strength

## Abstract

Soybean meal-based adhesives are attractive potential environmentally friendly replacements for formaldehyde-based adhesives. However, the low strength and poor water resistance of soybean meal-based adhesives limit their practical application. This study was conducted to develop a natural fiber-reinforced soybean meal-based adhesive with enhanced water resistance and bonding strength. Pulp fiber (PF), poplar wood fiber (WF), and bagasse fiber (BF) were added as fillers into the soybean meal-based adhesive to enhance its performance via hydrogen bonding between the PF and the soybean meal system. The enhanced adhesive exhibited a strong crosslinking structure characterized by multi-interfacial interactions wherein PF served as a bridging ligament and released residual stress into the crosslinking network. The crosslinked structure and improved interfacial interactions were confirmed by Fourier transform infrared (FTIR) spectrophotometry, thermogravimetric analysis (TGA), and scanning electron microscopy (SEM) measurements. Plywood bonded with 4 wt % PF-containing soybean meal-based adhesive exhibited a wet shear strength (1.14 MPa) exceeding that of plywood bonded with the control group by 75.4% due to the stable crosslinking network having efficiently transformed stress and prevented the permeation of water molecules.

## 1. Introduction

Soybean meal (SM)-based adhesive is a noteworthy substitute for formaldehyde-based adhesives due to its environmental friendliness and biodegradability [1,2,3,4]. The inherently low strength and low water resistance of SM-based adhesives, however, hinders its practical application [5,6,7]. Many chemical/physical modification methods have been used to strengthen and waterproof SM-based adhesives such as soy protein structure modification [8], crosslinking agent modification [9,10], and organic or inorganic blending [11,12,13]. In the soy protein structure modification method, soy protein molecules are unfolded after using denaturation agents. The organic or inorganic blending method allows mixing of the inorganic phases with the organic phases to increase the strength of the matrix. However, the enhancement effect of these two methods is not obvious. Crosslinking agent modification may be a particularly effective method for improving the water resistance and mechanical strength of SM-based adhesives. For example, epoxy compounds, natural gellan, and genipin have been successfully employed as modifying crosslinkers [5,14,15]. Introducing a crosslinking agent greatly increases the cost of the adhesive, while also increasing its brittleness.

Fiber-reinforced composites have attracted much attention because fibers can increase the strength and toughness of composites. Glass fiber and ceramic fiber can be added to adhesives as fillers for this purpose [16,17,18], but they are expensive and non-degradable; some chemical fibers are even harmful to the human body. Natural fibers are widely used to strengthen composite materials as they are low cost, renewable, lightweight, and flexible, and have good mechanical properties, unique acoustic, and thermal insulating performances, and show less safety and health concerns (no skin irritations) [16,17,18]. Kenaf fiber [19], for example, enhances the mechanical properties of composites but adding it is a relatively complex and expensive process which is ill-suited to industrial applications. Researchers have added cheaper natural fibers (e.g., pulp fiber, poplar wood fiber, bagasse fiber, hemp fiber, and flax fiber) to composites to successfully increase their strength and elasticity as well.

Pulp fiber (PF) is a type of natural fiber that has high elasticity, strong toughness, rich yields, and a favorable strength-to-weight ratio. It resembles spider silk—it is not easy to age and tends to react with other reagents. Yang et al. used PF to effectively enhance the tensile and bending strength of polylactic acid composites [20]. Natural poplar wood fiber (WF) also has relatively high strength. Nourbakhsh et al. added PF and several other fibers to nanotube composites and found that WF outperformed the other fibers [21]. Bagasse fiber (BF) [22], a waste product from sugar processing, has also been used to successfully reinforce the strength of cardanol-formaldehyde composites.

In this study, inspired by reinforced-concrete structures and the potential added value of biomass materials, a value-added, environmentally friendly, high-strength, and cost-effective resin which contains natural fiber fillers was explored. Firstly, three kinds of natural fibers were pretreated with alkali and then added to SM with a green cross-linking agent. A crosslinking network structure formed in the SM-based adhesive system which can be attributed to the physiochemical interaction between soy molecules and natural fibers. The chemical structures of natural fibers and SM-based adhesives were analyzed, and the mechanical properties, water resistance, micromorphology, and thermal stability were determined as discussed below.

## 2. Experimental Procedures

### 2.1. Materials

SM powder, with a 45.2% soy protein content (5.0% moisture content, 6.46% ash, 0.56% fat, and 38.12% carbohydrate) was purchased from Xiangchi Grain and Oil Company (Shandong, China) and milled to 200 mesh in a laboratory grinder. PF was purchased at Yangrun Trading Co., Ltd. (Dalian, China). WF was purchased from the Hongren Mineral Processing Plant (Shijiazhuang, China), and were chopped into portions of approximately 4–5 mm in length. BF was purchased from the Aigou Agricultural Products Department (Nanning, China) and then sifted into fibers of 4–5 mm in length. The crosslinking agent solution (69% solid content) was bought from Tianjin Heowns Biochem Co., Ltd. (Tianjin, China). Other chemical reactants were purchased from Beijing Chemical Reagents Co., Ltd. (Beijing, China).

### 2.2. Pre-Treatment of Natural Fibers

The three types of fibers (PF, WF, and BF) were each alkali pre-treated in the same manner. The fiber (40 g) was placed into NaOH solution (5%) then held in a high temperature sealed reactor for 2.5 h with mechanical stirring. After cooling, the treated fibers were washed with distilled water to neutralize the pH, then dried in an oven at 80 °C to a constant weight.

### 2.3. The SM-Based Adhesive Preparation

The CSM adhesive was made from the SM-based adhesive only modified by the crosslinking agent. Thirty grams of SM and 0.87 g of the crosslinking agent were dispersed sequentially in distilled water (70 g) under constant stirring for 30 min at 20 °C. The ratio of crosslinking agent weight to the SM weight was 1:50.

PCSM adhesives (PCSM-1,2,3); CSM adhesives modified by PF, were made by uniformly dispersing a predetermined amount of PF (2, 4, and 6 wt % rate of PF to SM weight) in the CSM (100 g) adhesive, and then stirring the mixture vigorously for 10 min at 20 °C. WCSM adhesives (WCSM-1,2,3); CSM adhesives modified by WF, and BCSM adhesives (BCSM-1,2,3), CSM adhesives modified by BF, were prepared similarly.

The modified SM-based adhesives with crosslinking agents (CSM adhesive) and PCSM, WCSM, and BCSM adhesives were prepared according to the formulations shown in Table 1.

### 2.4. Triple-Layered Plywood Specimen Preparation

The as-prepared adhesives were used to prepare three-layered plywood specimens at a spreading rate of 180 g/cm^2^ for each layer. The three-layered plywood was then hot-pressed at a temperature of 120 °C under a pressure of 1 MPa for 315 s (4.5 mm thick plywood) [23]. The obtained samples were stored at 20 °C and 60% relative humidity for 12 h before further testing.

### 2.5. Characterization of PF, WF, BF, and the Adhesive Samples

#### 2.5.1. ATR-FTIR Spectroscopy

The samples of the modified SM-based adhesives were completely cured in an oven (120 ± 2 °C) until reaching a constant weight and then ground into powder (200 mesh). The ATR spectra tests of the modified SM-based adhesives were observed on a Nicolet 7600 spectrometer (Nicolet Instrument Corp., Madison, WI, USA) equipped with an ATR accessory. The spectra were recorded over the range of 4000–650 cm^−1^ with a 4 cm^−1^ resolution using 32 scans [24].

#### 2.5.2. Thermogravimetric Analysis

The thermal stabilities of CSM/PCSM/WCSM/BCSM adhesives were recorded respectively on a TGA instrument (TA Q50, WATERS Company, New Castle, DE, USA). About 6 mg of adhesive sample powder was weighed in a platinum cup, then heated from 25 to 610 °C at a rate of 10 °C min^−1^ in a constant nitrogen atmosphere (100 mL min^−1^) [25]. Changes in weight were recorded throughout the whole process.

#### 2.5.3. Scanning Electron Microscopy

A Hitachi S-3400N (Hitachi Science System, Ibaraki, Japan) SEM was used to observe the morphologies of the fiber surface and the fractured cross-sections of the CSM/SM-based adhesives modified by the three types of fibers. Adhesive samples were completely cured in an oven (120 ± 2 °C), then fractured into several pieces to obtain cross-sections. The fiber surfaces and adhesive cross-sections were coated with 10 nm Au/Pd film before microscopy [26].

#### 2.5.4. Apparent Viscosity Measurement

The viscosity of the CSM/PCSM/BCSM/WCSM adhesives was measured using a rheometer with a parallel plate fixture (20 mm diameter). The distance was set to 1 mm, and the spinning rate was 2 rpm during the measurements. Six replicate measurements were performed for each kind of sample.

#### 2.5.5. Residue Rate Test

The residue rates of the adhesives were determined by gravimetric analysis. Adhesive samples were dried in an oven set at 120 ± 2 °C until a constant weight (m_1_), then immersed in water for six h in an oven s at 60 ± 2 °C and dried at 105 ± 2 °C for three h to a constant weight (m_2_). The residue rate was calculated as m_1_/m_2_ and reported as a percentage. All of the measurements were made in triplicate.

#### 2.5.6. Dry and Wet Shear Strength Measurements

The dry and wet shear strengths of the interior used plywood (Type II plywood, ≥0.7 MPa) and were tested on an electronic universal testing machine and evaluated according to the China National Standard GB/T 17657-2013. Each plywood panel was cut into 14 plywood specimens (100 mm × 25 mm). The dry shear strengths of the specimens were tested at an operating speed of 20.0 mm min^−1^ on the testing machine. The plywood specimens were immersed in water at 63 °C for 3 h, dried for 10 min at room temperature, and tested for the wet shear strength. The speed of the crosshead was 10 mm min^−1^. The values were calculated as follows:

(1) $$\mathrm{Dry}/\mathrm{wet}\;\mathrm{shear}\;\mathrm{strength}\left(\mathrm{MPa}\right)=\frac{\mathrm{Force}\;\left(N\right)}{\mathrm{Gluing}\;\mathrm{area}\;\left({\mathrm{mm}}^{2}\right)}$$

#### 2.5.7. Statistical Analysis

The differences among specimens were compared by Duncan’s multiple range test at *p* = 0.05. Experiments were repeated at least six times, and the variance was analyzed accordingly [27].

## 3. Results and Discussion

### 3.1. Structural Analysis of the Adhesives

#### 3.1.1. ATR-FTIR Spectroscopy Results

**Figure 1:**
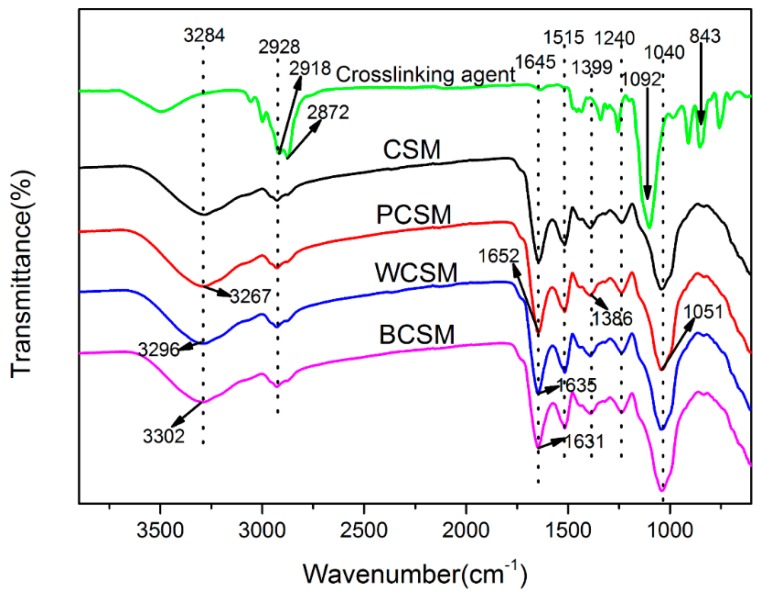
ATR spectra of CSM, PCSM, WCSM, and BCSM adhesives.

The possible structures between different fibers and SM-based adhesives were investigated by ATR-FTIR spectroscopy. ATR-FTIR spectra of the cured CSM adhesive and hybrid adhesives are shown in Figure 1.

In the spectra of the CSM adhesive, a broad absorption band in the range of 3500–3200 cm^−1^ corresponds to the bending vibrations of free and bound O–H and N–H groups. The peak observed at 2928 cm^−1^ can be attributed to the symmetric and asymmetric stretching vibrations of the –CH_2_ groups [28]. The absorption bands near 1399 and 1040 cm^−1^ can be respectively attributed to the stretching vibration of COO– and C–O. Dominant peaks were observed at approximately 1645, 1515, and 1240 cm^−1^ as attributed to C=O stretching (amide I), N–H deformation (amide II), and C–N stretching and N–H vibration (amide III), respectively [29]. In the crosslinking agent spectrum, the peaks at 2918 and 2872 cm^−1^ corresponded to the vibration of –CH_2_, and the peaks at 1092 and 843 cm^−1^ were attributed to the C–O–C and free epoxy group skeleton vibrations, respectively. The peak at 843 cm^−1^ of the epoxy groups disappeared in all the SM-based adhesives. This outcome was expected because the crosslinking agent could form covalent bonds/hydrogen bonds with the amino group of the SM by the ring opening reaction [28].

**Scheme 1:**
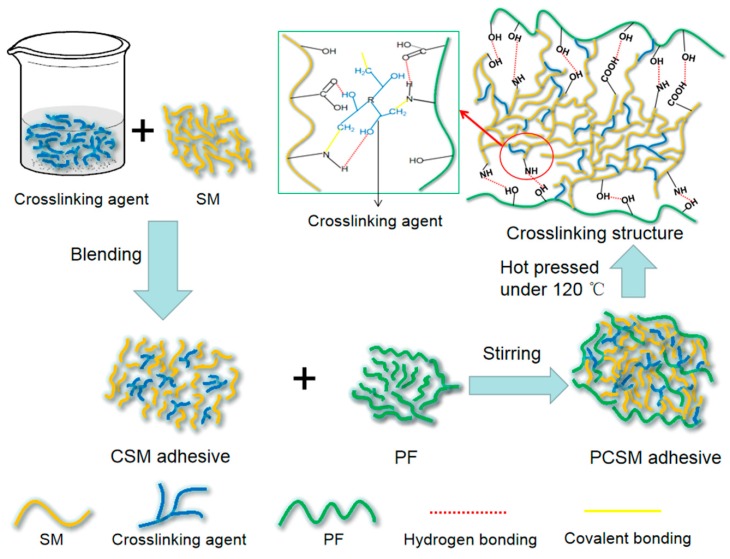
Schematic illustration of the PCSM adhesive crosslinking mechanism.

After adding PF, the hydroxyl peak shifted to a lower wavenumber in the spectrum of the PCSM adhesive; that of PCSM shifted from 3284 to 3267 cm^−1^ indicating that the –OH groups in the PF participated in the adhesive system via intermolecular hydrogen bonding [30]. In addition to the above, the C=O groups (1645 cm^−1^) of amide I shifted toward a higher wavenumber (PCSM was at 1652 cm^−1^) in the spectrum of the PCSM, which further indicates the formation of hydrogen bonds between the fibers and adhesive molecules [28]. The peaks of –C–NH_2_ bending (at 1040 cm^−1^) also moved to higher frequencies; that of PCSM shifted to 1051 cm^−1^. The peak at 1399 cm^−1^ attributed to the COO– groups shifted to lower frequencies; that of PCSM shifted to 1386 cm^−1^, which indicates the construction of multiple reactions between the fibers and adhesive molecules [4,31]. The addition of PF enhanced the mechanical strength of the adhesive. A crosslinking model of the PCSM adhesive is provided in Scheme 1.

Interestingly, the hydroxyl peak shifted to higher wavenumbers, and C=O group peaks shifted to lower wavenumbers in the spectra of the WCSM and BCSM adhesives, which means that there was less hydrogen bonding than in the PCSM adhesive. This also indicates that the addition of WF and BF reduced the hydrogen bonds in the adhesive and did not effectively enhance the material’s mechanical strength. Dense crosslinking structures were formed by hydrogen bonding between PF and SM in the PCSM adhesive, which enhanced the strength and water resistance of adhesive. The WF and BF did not form the effective crosslinking structures with the SM-based system and destroyed the structures of the material itself, which was detrimental to the strength of the adhesive.

#### 3.1.2. Thermogravimetric Analysis

**Figure 2:**
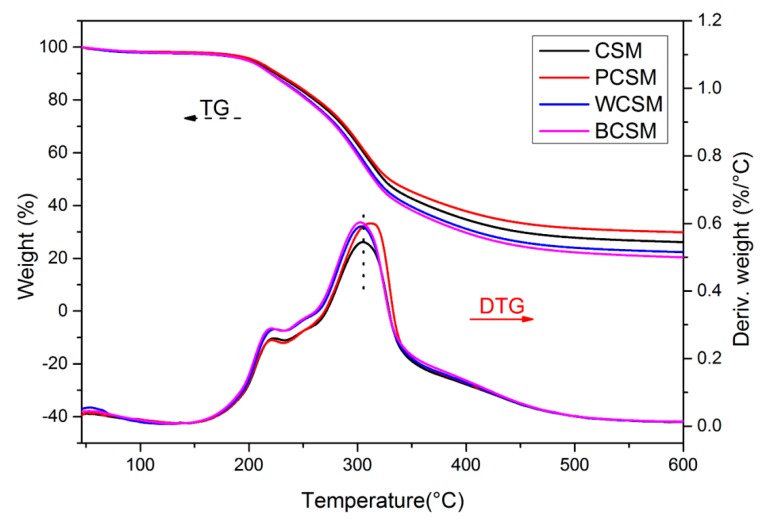
Thermogravimetry and derivative thermogravimetry curves of CSM, PCSM, WCSM, and BCSM adhesives.

To further investigate the chemical structures between different fibers and SM-based adhesives, we examined their thermal behavior by TGA as shown in Figure 2. The thermal degradation data is also listed in Table 2. The thermal degradation process can be roughly divided into two stages: 100–250 °C (stage 1), and 250–500 °C (stage 2). Two peaks were observed for the CSM adhesive in the DTG curve. The first peak (222 °C) at stage 1 can be attributed to the loss of micromolecules and decomposition of unstable chemical bonds [32]. The second peak (305 °C) at stage 2 occurred due to the skeleton structure degradation of the adhesive [33]. Some weight was lost before the first stage as the protein broke down and the water evaporated. Further heating beyond the second stage caused the C–C, C–N, and C–O linkages to break [34].

After adding WF and BF, the peak value at stage 2 shifted to a lower temperature compared to the CSM adhesive in the DTG curve. The carbon residue rate also reduced significantly in the TG curve, which indicated that the thermal stability of WCSM and BCSM adhesives was lower than that of CSM. This is likely because WF and BF broke the crosslinking network of the adhesive and weakened the interface interaction. After incorporating the PF modifier, the peak value at stage 2 of the PCSM shifted to a higher temperature in the DTG curve compared to the CSM curve where the carbon residue rate of the PCSM also increased significantly compared to that of the CSM. This suggested that the thermal stability of the PCSM adhesive was higher than that of the CSM adhesive, which may be attributable to the construction of multiple interactions and the increase in crosslinking robustness after PF addition.

We also found that the peak value of PCSM was the lowest and its carbon residue rate was the highest among all SM-based adhesives. This suggested that the thermal stability of the adhesive with PF was the best among all samples. Proper PF loading appeared to result in a firm crosslinking structure that enhanced the thermal stability of the adhesive. The dense crosslinking structure, enhanced thermal stability, and superiority of PCSM adhesives over WCSM and BCSM adhesives as-evidenced by TGA were also supported by the ATR-FTIR spectroscopy results.

#### 3.1.3. Micromorphological Analysis

**Figure 3:**
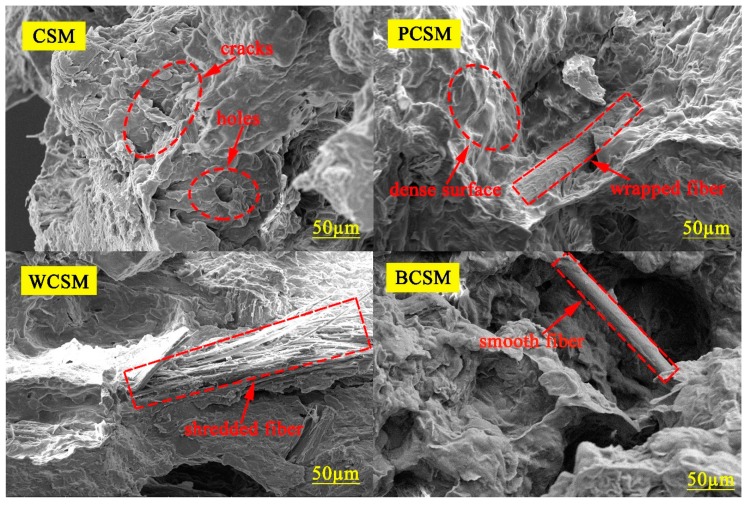
SEM images of surface morphology for cured CSM, PCSM, WCSM, and BCSM adhesive samples.

The surface micromorphological analysis was applied to observe the structures in the matrix among different fibers and SM-based adhesives. The fracture surfaces of the cured CSM adhesives and hybrid adhesives were observed by SEM, as shown in Figure 3. The CSM adhesive (Figure 3(CSM)) showed a relatively loose and discontinuous fracture surface with several holes and cracks. These defects were caused by the amorphous features of the CSM adhesive, which is easily penetrated by water [35]. After adding WF and BF (WCSM, BSCM), the cracks and holes remained. As shown in Figure 3(WCSM), some WF were shredded in the adhesive. In effect, the WF did not establish an effective crosslinking structure with the adhesive layer resulting in its destruction under stress. The lack of an effective crosslinking structure may have been due to the relatively loose surface of the WF.

In Figure 3(BCSM), a small amount of BF detached from the adhesive surface. This reflects the weak interactions between the fibers and resin—the fiber did not play a role in strengthening the adhesive. The weak interaction may be due to the fact that the BF surface was porous and absorbed water and the steam generated during hot-pressing destroyed the interaction between the BF and the resin.

Fewer holes and cracks were observed after PF addition, and the fracture surface became more compact. In other words, the mechanical properties of the adhesive were improved by PF. Few fibers were pulled out in the PCSM, which reflected a strong interface between the matrix and the PF. This interaction was closely related to the favorable surface morphology of PF. In the Figure 3(PCSM) sample, the PF acted like twisted steel embedded in the resin. When microcracks propagated through the PF in the resins, the PF acted as a bridging ligament to deflect it as the strength of the fiber was much larger than that of the adhesive matrix. The PF effectively released the residual stress and inhibited the extension of microcracks [36], thus resulting in improved mechanical performance.

**Figure 4:**
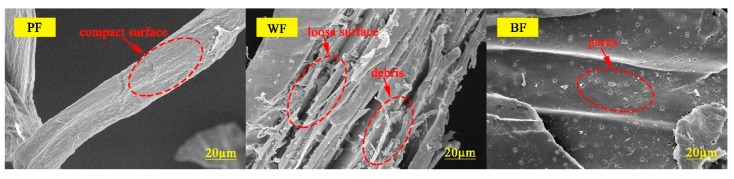
SEM images of the micro-topographies for PF, WF, and BF.

The micro-topographies of PF, WF, and BF were next observed to determine why different fibers caused different adhesive surface morphologies (Figure 4). As shown in Figure 4(WF), the WF had a loose surface covered in debris. These fibers were likely to reduce interface interactions and resulted in poor interface contact. The BF surface also appeared to be very porous (Figure 4(BF)); the pores absorbed large amounts of water and produced a great deal of water vapor during the heating process. By contrast, the surface of the PF was very compact (Figure 4(PF)). The PF has a larger length-to-diameter ratio than the other fibers (the “twisted steel” property mentioned above), which facilitated strong interactions with other molecules in the adhesive. The surface morphology of PF allowed for a solid crosslinking structure that improved the strength of the adhesive.

The micromorphological analysis results were effectively validated by the above analysis of PF, WF, and BF micro-topographies. The crosslinking structures of the adhesives with PF were denser—PF served as a bridging ligament and released the residual stress in the crosslinking structure, which effectively improved the adhesive strength and water resistance of the material. The WF surface was too loose and the BF surface too porous to effectively construct the desired adhesive crosslinking network. The micromorphological analysis also supports the ATR-FTIR spectroscopy and TGA analysis results.

#### 3.1.4. Apparent Viscosity Analyses

The apparent viscosity of the adhesive largely reflects the penetration and flow capacity of the adhesive. The too high viscosity of the adhesive would cause difficulty in distributing the adhesive evenly on the wood surfaces, while the too low viscosity of the adhesive would lead to over-penetration. Therefore, the adhesive strength could be reduced by either a too high or too low viscosity of the adhesive. As a well-known rule of thumb, the optimal values of viscosity should range from 5000 to 25,000 MPa s for composite adhesives. As shown in Table 3, the viscosity of CSM was low, which proved that the crosslinking density of CSM was low, which would cause the problem of over-penetration. After the 0–4 wt % PF addition, the viscosity of the adhesive was increased. This behavior proved that the PF had multiple interactions with the adhesive molecules and strengthened the crosslinking network. The reinforcement of the crosslinking network reduced the flow capacity of the adhesive. However, the excessive addition of PF resulted in the decrease of adhesive viscosity, which was because the excessive PF broke the crosslinking network. It can be observed that the viscosity of the adhesive decreased after adding WF and BF, which can be because WF and BF did not effectively bind with the adhesive molecules.

#### 3.1.5. Residue Rate Analyses

**Figure 5:**
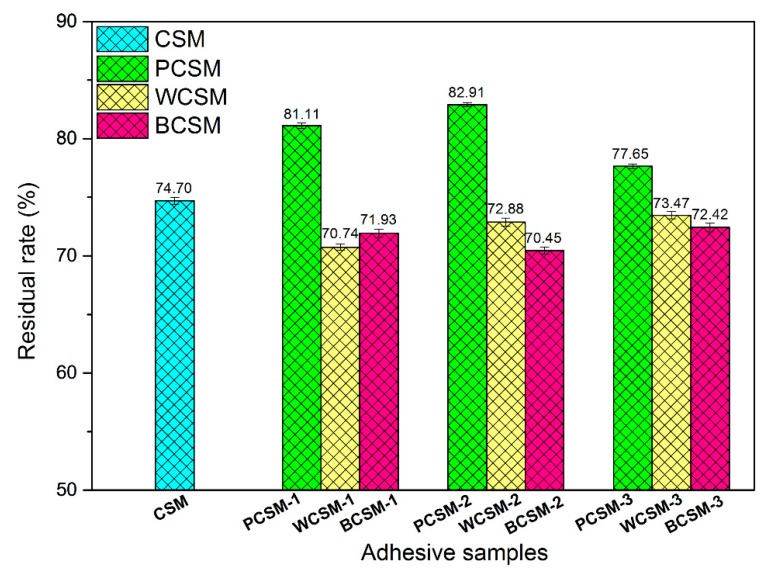
The residue rate of CSM and PCSM, WCSM, Band CSM adhesive samples.

The residue rate is an important parameter in evaluating the hydrolysis stability of adhesives. It reflects the density of the crosslinked structure in the adhesive to a certain extent, as well as the detailed structures of different fibers and SM-based adhesives. As shown in Figure 5, the CSM adhesive has a residue rate of 74.70%. The addition of WF to the CSM adhesive decreased the residue rate from 74.70% to 70.74% (WCSM-1). The residue rate of the adhesive decreased from 74.70% to 70.45% (BCSM-2) after the addition of BF. These characteristics may be attributable to the porous and loose surfaces of WF and BF preventing crosslinking between the fibers and the adhesive molecules.

Conversely, the addition of 0–4 wt % PF into the CSM adhesive improved the residue rate of the latter from 74.70% to 82.91%. The residue rate of PCSM-2 was the highest among the samples, exceeding that of CSM adhesives by 11%. PF participated in the crosslinking reaction to afford multiple interactions with the adhesive molecules, which improved the crosslinking density and water resistance of the adhesive and thus the residue rate. However, the continued increase in fiber addition to 6 wt % (PCSM-3) decreased the adhesive residue rate to 77.65%. If the adhesive crosslinking network was damaged by excessive fibers, the water resistance of the adhesive would decrease. The appropriate PF addition can effectively produce a crosslinking structure with other molecules in the adhesive via multi-interfacial interactions, which improves the water resistance of the adhesive. The addition of WF and BF, as discussed above, did not result in the construction of a dense crosslinked structure thus reducing the water resistance of the adhesive. The residue rate analysis results are in accordance with the results from previous tests.

### 3.2. The Effect of Different Fibers on SM-Based Adhesive Mechanical Properties

#### Dry and Wet Shear Strength Measurements

**Figure 6:**
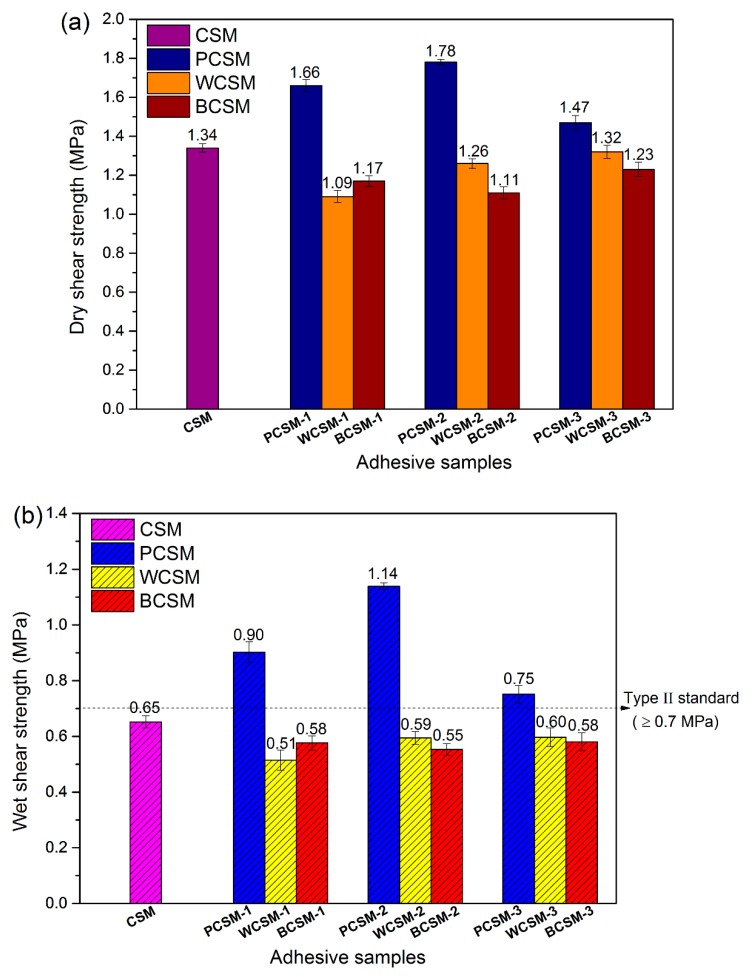
Dry (**a**) and wet (**b**) shear strengths of CSM, PCSM (1–3), WCSM (1–3), and BCSM (1–3) adhesives.

The dry and wet shear strengths of plywood samples fabricated with different adhesive formulations were assessed to further explore their mechanical properties, as shown in Figure 6. The dry and wet shear strengths of the CSM adhesive were measured at 1.34 and 0.65 MPa, respectively. The wet shear strength did not meet the interior use requirement (≥0.7 MPa) according to the China National Standard (GB/T 9846.3-2004). The dry and wet shear strengths of plywood bonded with PCSM adhesives (PCSM-1, PCSM-2, and PCSM-3) were significantly higher than those of the CSM adhesive, and do meet the interior use requirement. When 4% PF was added to the adhesive (PCSM-2), the dry and wet shear strengths reached a peak of 1.78 and 1.14 MPa, respectively, marking an increase of 56.1% (dry), and an increase of 75.4% (wet) compared to the CSM adhesive.

The enhancements discussed above could be explained according to two main factors. First, PF formed physical and chemical interactions with the adhesive molecules [37,38]. The strong interactions ensured the construction of a stable crosslinking network and enhanced the ability of the plywood to resist moisture intrusion. Second, PF played a bridging role and released residual stress in the crosslinking network of the adhesive, allowing the plywood bonded by the PF-enhanced SM-based adhesive to bear more stress. The excessive addition of PF (6 wt %) reduced the dry and wet shear strengths of the PSCM adhesives, likely because an excess of fiber led to an increase in harmful friction between the adhesive components which damaged the crosslinking network.

We also found that the dry and wet shear strengths of the WCSM and BCSM adhesives were lower than those of the CSM adhesive in general due to the loose and porous structural characteristics of WF and BF. The loose structure in the WCSM adhesives caused the crosslinking unstable and was easy to be broken by tension. The pores in the BCSM adhesives reduced the contact area for other substances and resulted in the fiber absorbing a lot of water. Bad bonding was produced during hot-pressing which resulted in a decrease in the residue rate and strength.

The qualities of the PF in terms of crosslinking structure, strength, and water resistance—particularly compared to the mechanical effects of WF and BF addition—suggest that PF can be used to fabricate high-performance hydrogels, bio-films, and bio-adhesives.

## 4. Conclusions

In this study, a new formulation of a sustainable, biomass-based, and fiber-reinforced adhesive was established. Three kinds of fibers (PF, WF, and BF) were added as fillers to the crosslinking agent modified adhesive CSM. We found that adding PF effectively enhanced the mechanical strength of the adhesive, while WF and BF reduced the mechanical strength of the adhesive. The wet shear strength reached a maximum of 1.14 MPa after the addition of 4% PF, marking a 72.3% increase over that of the CSM adhesive. The PF-containing hydroxyl groups served as reinforcing material for interfacial hydrogen bonds and a crosslinking network between PF and adhesive molecules, as confirmed by FTIR and SEM analyses. These multiple interactions contributed to the significant improvement in the adhesion, water resistance, and thermal stability of the adhesive.

## Figures and Tables

**Table 1:** Experimental Formulations of SM-Based Adhesives (Since the amount of SM, deionized water and the crosslinking agent is the same in all samples, they are not listed in the table).

Sample	Fiber
CSM	0
PCSM-1	2 wt % of PF
PCSM-2	4 wt % of PF
PCSM-3	6 wt % of PF
WCSM-1	2 wt % of WF
WCSM-2	4 wt % of WF
WCSM-3	6 wt % of WF
BCSM-1	2 wt % of BF
BCSM-2	4 wt % of BF
BCSM-3	6 wt % of BF

**Table 2:** Thermal degradation of CSM and PCSM, WCSM, and BCSM adhesives.

Sample	T_max1_ (°C)	T_max2_ (°C)	Residual Mass (wt %) at 600 °C
CSM	220.51	304.53	26.14
PCSM	223.58	313.82	29.92
WCSM	216.98	301.75	22.48
BCSM	216.35	297.61	20.38

**Table 3:** The apparent viscosity of CSM and PCSM, WCSM, and BCSM adhesives.

Sample	Viscosity (MPa s)
CSM	9524
PCSM-1	14,654
PCSM-2	23,398
PCSM-3	22,268
WCSM-1	9029
WCSM-2	7901
WCSM-3	6502
BCSM-1	8168
BCSM-2	6945
BCSM-3	6201

## Nomenclature

SM	Soybean meal
CSM	SM-based adhesive only modified by the crosslinking agent
PCSM adhesives	CSM adhesives modified by pulp fiber
WCSM adhesives	CSM adhesives modified by poplar wood fiber
BCSM adhesives	CSM adhesives modified by bagasse fiber
ATR-FTIR	Attenuated total reflection–Fourier transform infrared spectroscopy
TGA	Thermogravimetry
DTG	Derivative thermogravimetry
